# Supplementary material for: Dinosaur biodiversity declined well before the asteroid impact, influenced by ecological and environmental pressures
Source: Nat Commun. 2021 Jun 29;12:3833. doi: 10.1038/s41467-021-23754-0 (PMC8242047; doi:10.1038/s41467-021-23754-0)
Supplement: Supplementary file 1 — Supplementary Information [file 41467_2021_23754_MOESM1_ESM.pdf]

Supplementary Information

Dinosaur biodiversity declined well before the asteroid impact, influenced by ecological and environmental pressures

Fabien L. Condamine[1]*, Guillaume Guinot[1], Michael J. Benton[2] & Philip J. Currie[3]

Affiliations

[1]*Institut des Sciences de l'Evolution de Montpellier (Université de Montpellier | CNRS | IRD | EPHE), Place Eugène Bataillon, 34095 Montpellier, France;*

[2]*Department of Earth Sciences, University of Bristol, Bristol BS8 1RJ, UK;*

[3]*Department of Biological Sciences, University of Alberta, Edmonton, AB T6G 2E9, Canada.*

These authors jointly supervised this work: Michael J. Benton, Philip J. Currie.

Correspondence (*): fabien.condamine@gmail.com

Supplementary Figure 1. Diversification and diversity dynamics of Late Cretaceous dinosaurs. The Bayesian RJMCMC model estimates speciation rate (**a**), time shifts in speciation rate (**b**), extinction rate (**c**), time shifts in extinction rate (**d**), net diversification rate (**e**, speciation minus extinction) rates, and species longevity in million years (**f**). Solid lines indicate mean posterior rates and shaded areas show 95% CI. As in the BDMCMC model, net diversification decreased and became negative ~76 Myrs ago (late Campanian). The vertical dashed red line indicates the Cretaceous-Palaeogene mass extinction (66 Myrs ago).

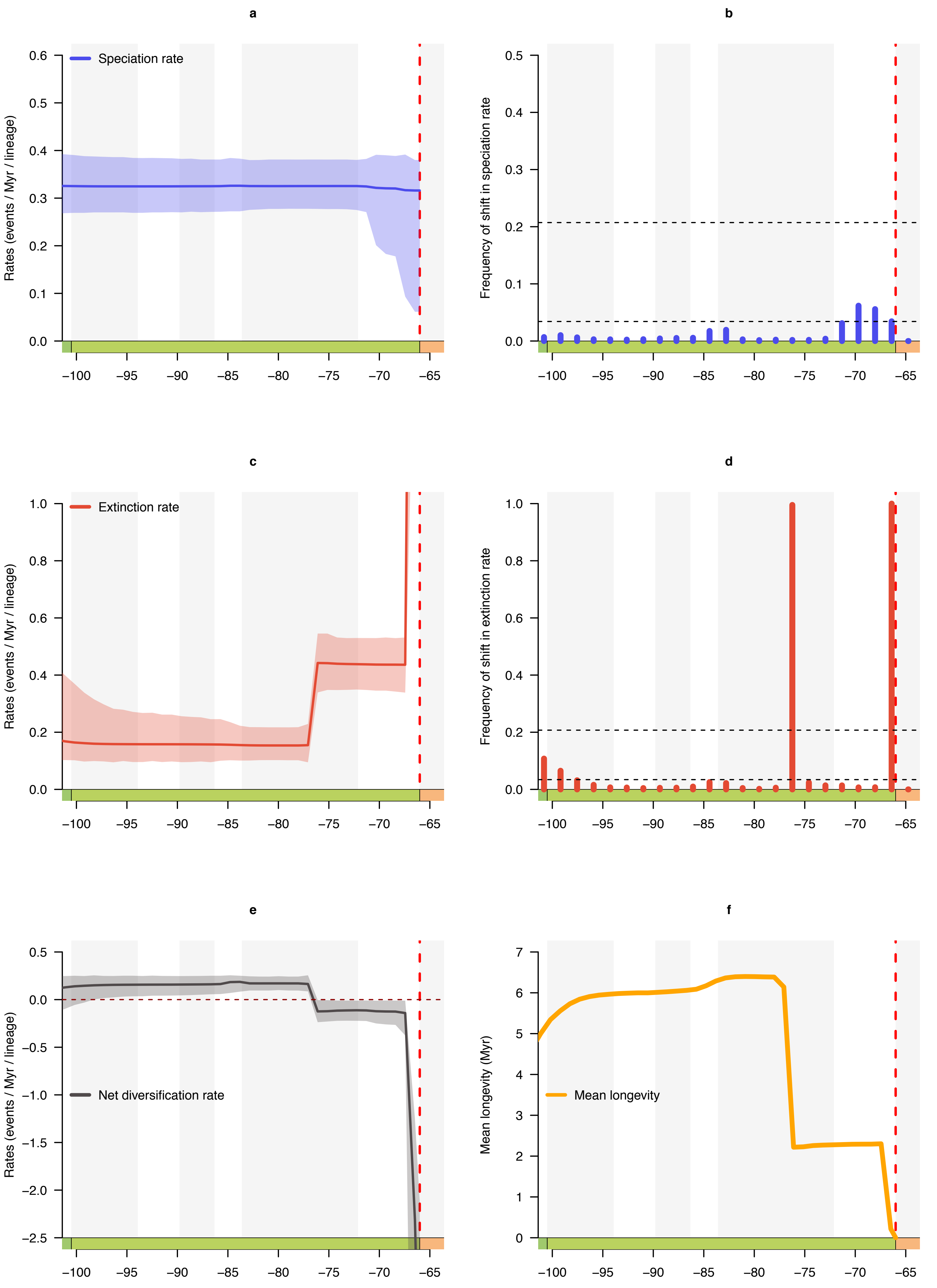
a
Speciation rate
Rates (events / Myr / lineage)
b
Frequency of shift in speciation rate
c
Extinction rate
Rates (events / Myr / lineage)
d
Frequency of shift in extinction rate
e
Net diversification rate
Rates (events / Myr / lineage)
f
Mean longevity
Mean longevity (Myr)
−100
−95
−90
−85
−80
−75
−70
−65

Supplementary Figure 2. Diversification and diversity dynamics of Late Cretaceous herbivorous dinosaurs. The Bayesian RJMCMC model estimates speciation rate (**a**), time shifts in speciation rate (**b**), extinction rate (**c**), time shifts in extinction rate (**d**), net diversification rate (**e**, speciation minus extinction) rates, and species longevity in million years (**f**). Solid lines indicate mean posterior rates and shaded areas show 95% CI. As in the BDMCMC model, net diversification decreased and became negative ~76 Myrs ago (late Campanian). The vertical dashed red line indicates the Cretaceous-Palaeogene mass extinction (66 Myrs ago).

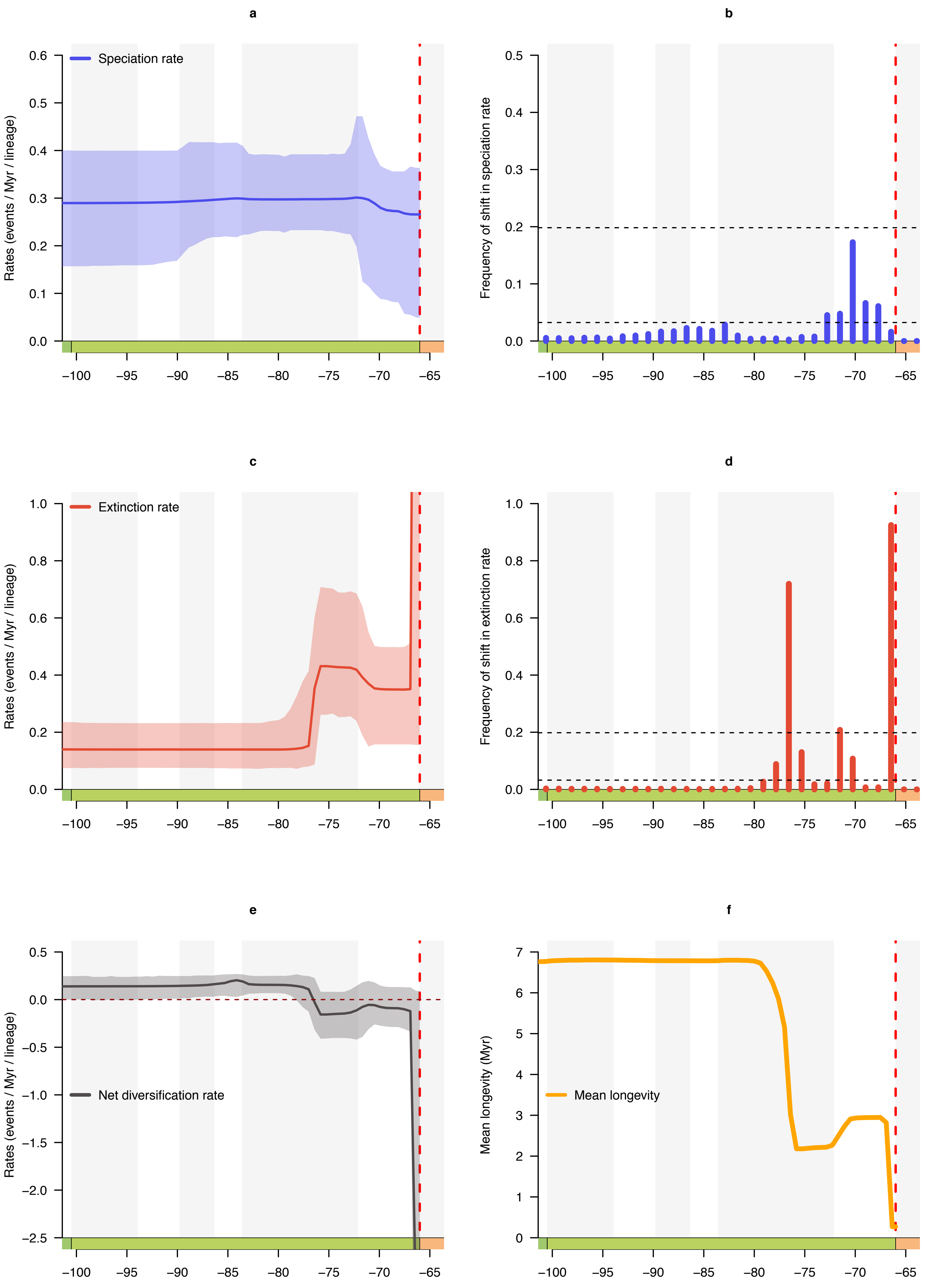
a
Speciation rate
Rates (events / Myr / lineage)
b
Frequency of shift in speciation rate
c
Extinction rate
Rates (events / Myr / lineage)
d
Frequency of shift in extinction rate
e
Net diversification rate
Rates (events / Myr / lineage)
f
Mean longevity
Mean longevity (Myr)
−100 −95 −90 −85 −80 −75 −70 −65

Supplementary Figure 3. Diversification and diversity dynamics of Late Cretaceous carnivorous dinosaurs. The Bayesian RJMCMC model estimates speciation rate (**a**), time shifts in speciation rate (**b**), extinction rate (**c**), time shifts in extinction rate (**d**), net diversification rate (**e**, speciation minus extinction) rates, and species longevity in million years (**f**). Solid lines indicate mean posterior rates and shaded areas show 95% CI. As in the BDMCMC model, net diversification decreased and became negative ~72 Myrs ago (early Maastrichtian). The vertical dashed red line indicates the Cretaceous-Palaeogene mass extinction (66 Myrs ago).

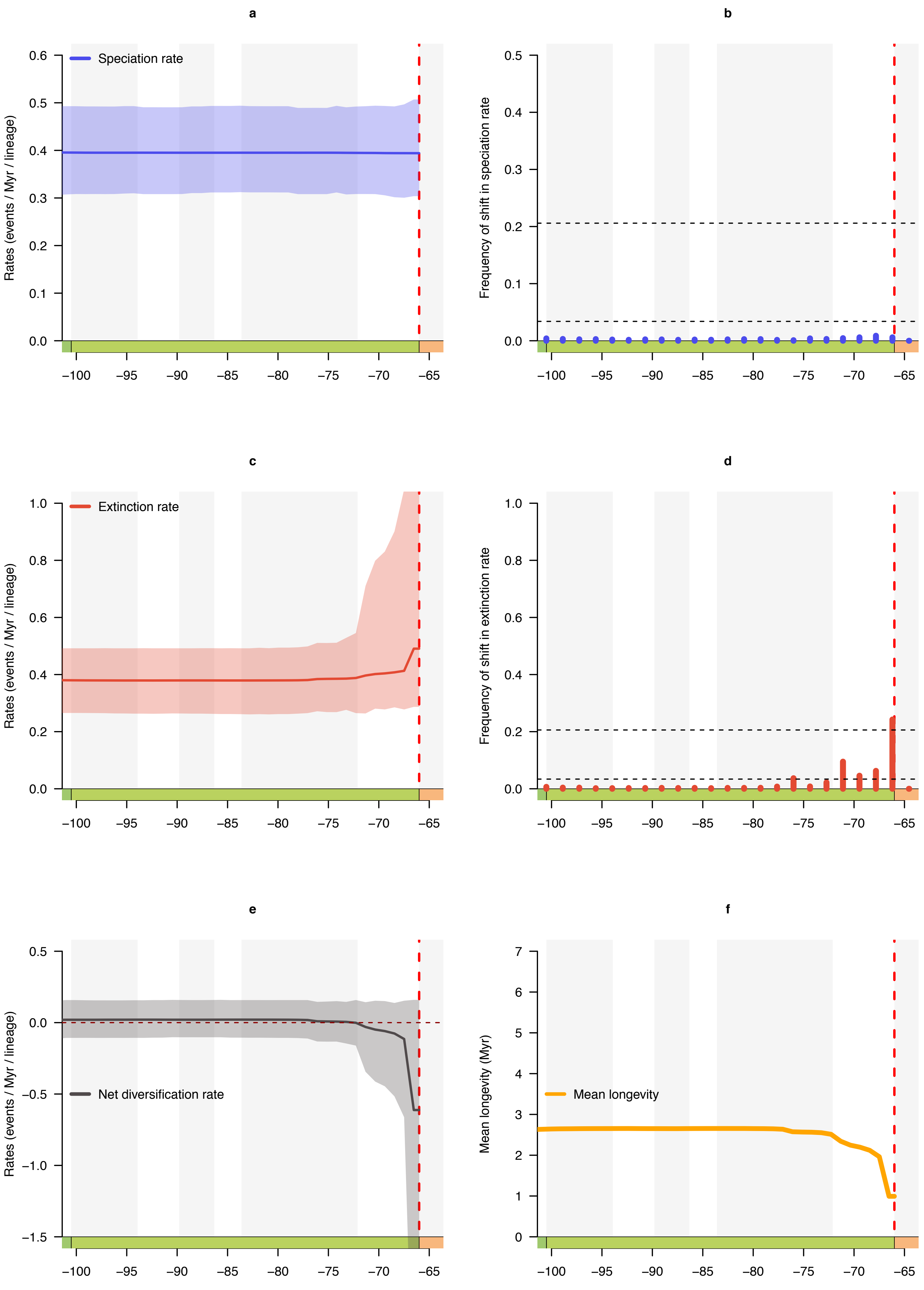
a
b
c
d
e
f
Speciation rate
Extinction rate
Net diversification rate
Mean longevity
Rates (events / Myr / lineage)
Frequency of shift in speciation rate
Frequency of shift in extinction rate
Mean longevity (Myr)
0.0
0.1
0.2
0.3
0.4
0.5
0.6
0.2
0.4
0.6
0.8
1.0
−1.5
−1.0
−0.5
1
2
3
4
5
6
7
−100
−95
−90
−85
−80
−75
−70
−65

Supplementary Figure 4. Diversification and diversity dynamics of Late Cretaceous dinosaurs. The Bayesian BDMCMC model estimates speciation and extinction rates (**a**), net diversification rate (**b**, speciation minus extinction) rates, and species diversity dynamics (**c**) for all dinosaur families at a global scale. The same results are shown for the New World dinosaurs (**d, e, and f**), and for the Old World dinosaurs (**g, h, and i**). Solid lines indicate mean posterior rates and shaded areas show 95% CI. The net diversification decreased and became negative in the late Campanian for the New World dinosaurs and in the early Maastrichtian for the Old World dinosaurs. Both the New World and Old World dinosaurs were in decline before the Cretaceous-Palaeogene (K-Pg) mass extinction (66 Myrs ago), represented by a vertical dashed red line. Asteroid icon made by Fabien Condamine.

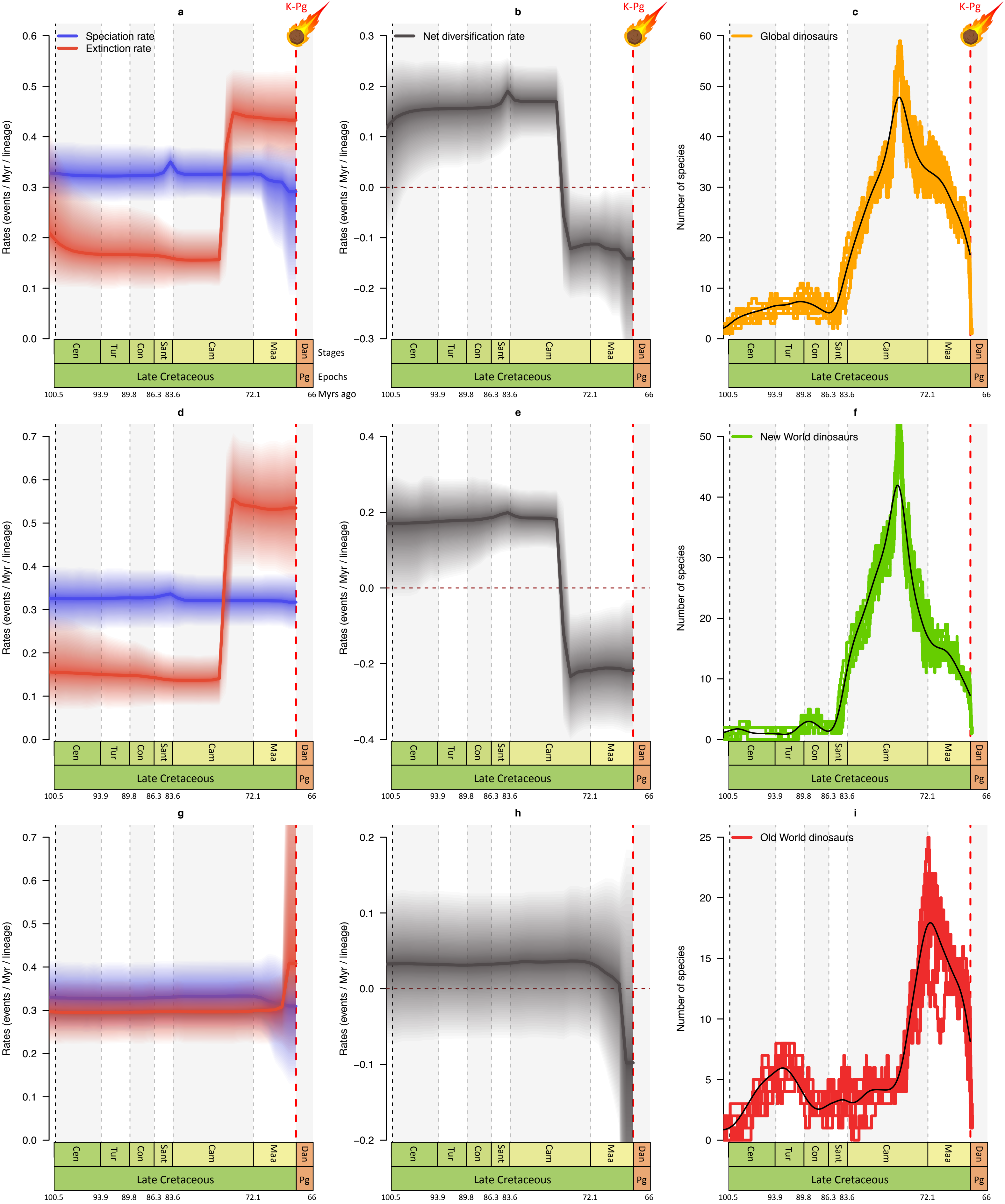
a
b
c
d
e
f
g
h
i
K-Pg
Speciation rate
Extinction rate
Net diversification rate
Global dinosaurs
New World dinosaurs
Old World dinosaurs
Rates (events / Myr / lineage)
Number of species
Cen
Tur
Con
Sant
Cam
Maa
Dan
Stages
Late Cretaceous
Pg
Epochs
100.5
93.9
89.8
86.3
83.6
72.1
66
Myrs ago

Supplementary Figure 5. Diversification and diversity dynamics of Late Cretaceous ankylosaurs. The Bayesian RJMCMC model estimates speciation rate (**a**), time shifts in speciation rate (**b**), extinction rate (**c**), time shifts in extinction rate (**d**), net diversification rate (**e**, speciation minus extinction) rates, and species longevity in million years (**f**). Solid lines indicate mean posterior rates and shaded areas show 95% CI. As in the BDMCMC model, net diversification decreased and became negative ~76 Myrs ago (late Campanian), mostly due to an increase of extinction rate. The vertical dashed red line indicates the Cretaceous-Palaeogene mass extinction (66 Myrs ago).

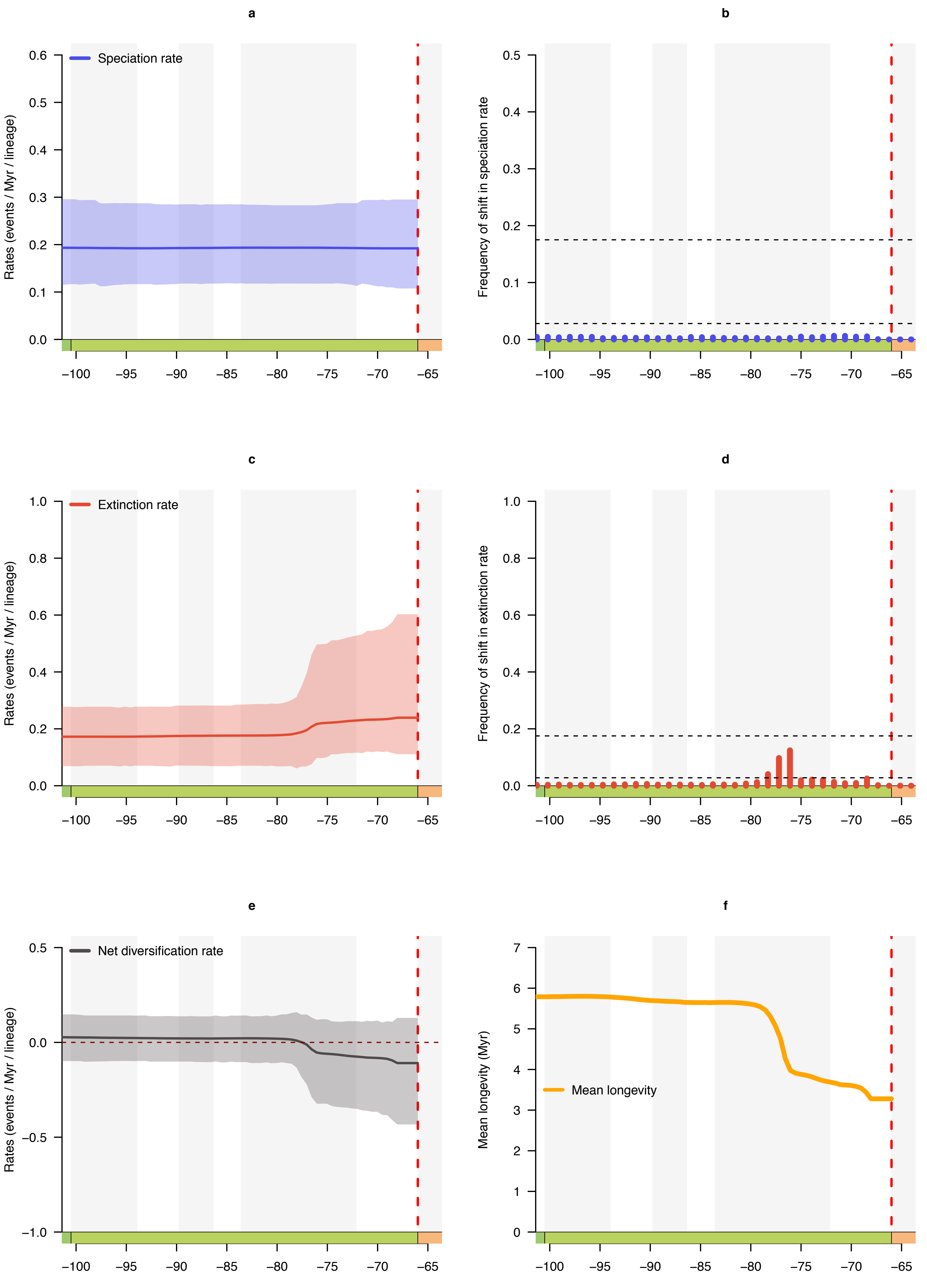
a
Speciation rate
Rates (events / Myr / lineage)
b
Frequency of shift in speciation rate
c
Extinction rate
Rates (events / Myr / lineage)
d
Frequency of shift in extinction rate
e
Net diversification rate
Rates (events / Myr / lineage)
f
Mean longevity
Mean longevity (Myr)

Supplementary Figure 6. Diversification and diversity dynamics of Late Cretaceous ceratopsians. The Bayesian RJMCMC model estimates speciation rate (**a**), time shifts in speciation rate (**b**), extinction rate (**c**), time shifts in extinction rate (**d**), net diversification rate (**e**, speciation minus extinction) rates, and species longevity in million years (**f**). Solid lines indicate mean posterior rates and shaded areas show 95% CI. As in the BDMCMC model, net diversification decreased and became negative ~76 Myrs ago (late Campanian), mostly due to an increase of extinction rate. The vertical dashed red line indicates the Cretaceous-Palaeogene mass extinction (66 Myrs ago).

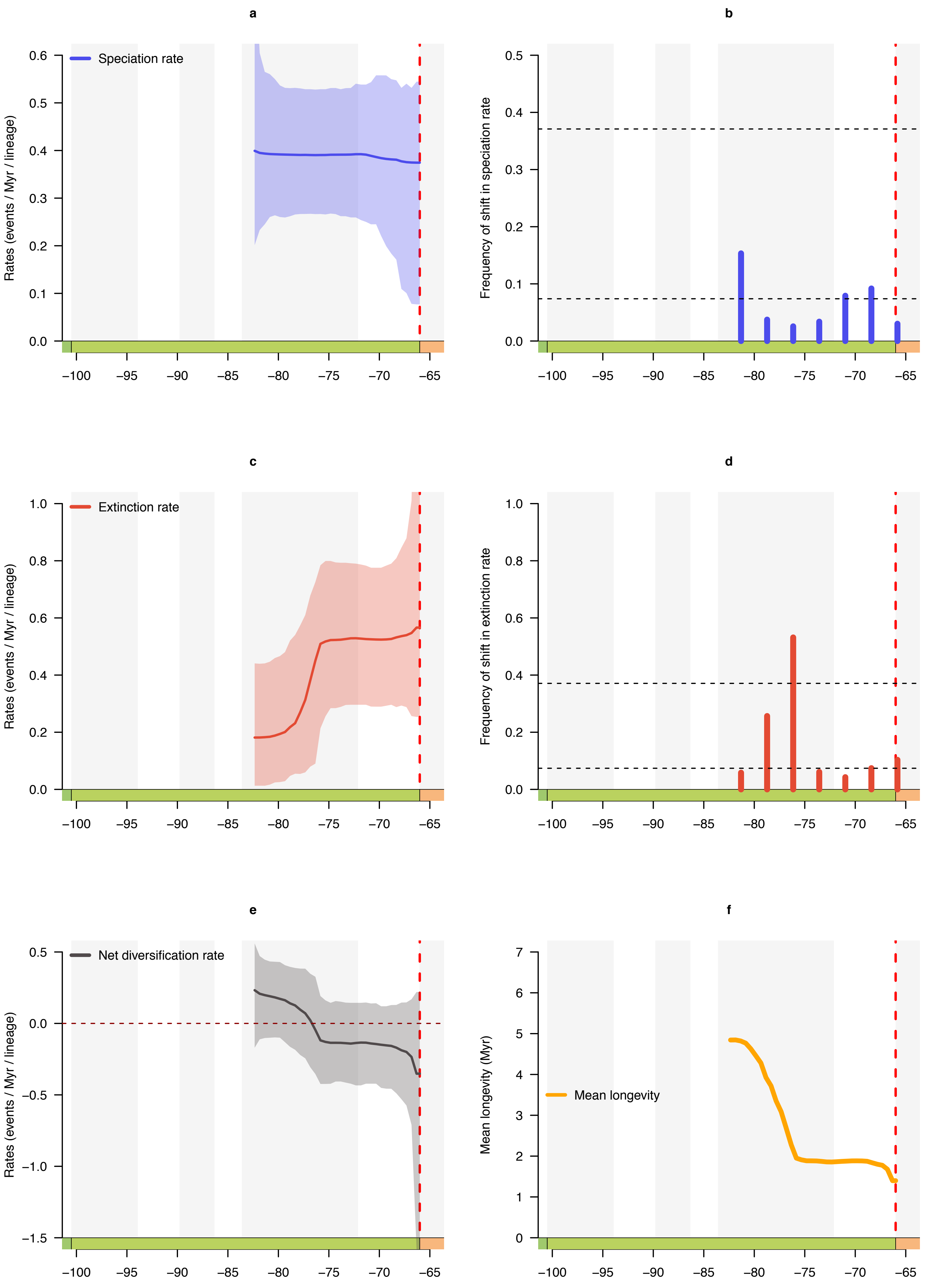

a
Speciation rate
Rates (events / Myr / lineage)
b
Frequency of shift in speciation rate
c
Extinction rate
Rates (events / Myr / lineage)
d
Frequency of shift in extinction rate
e
Net diversification rate
Rates (events / Myr / lineage)
f
Mean longevity
Mean longevity (Myr)

Supplementary Figure 7. Diversification and diversity dynamics of Late Cretaceous dromaeosaurs. The Bayesian RJMCMC model estimates speciation rate (**a**), time shifts in speciation rate (**b**), extinction rate (**c**), time shifts in extinction rate (**d**), net diversification rate (**e**, speciation minus extinction) rates, and species longevity in million years (**f**). Solid lines indicate mean posterior rates and shaded areas show 95% CI. As in the BDMCMC model, net diversification decreased and became negative ~72 Myrs ago (early Maastrichtian), mostly due to an increase of extinction rate. The vertical dashed red line indicates the Cretaceous-Palaeogene mass extinction (66 Myrs ago).

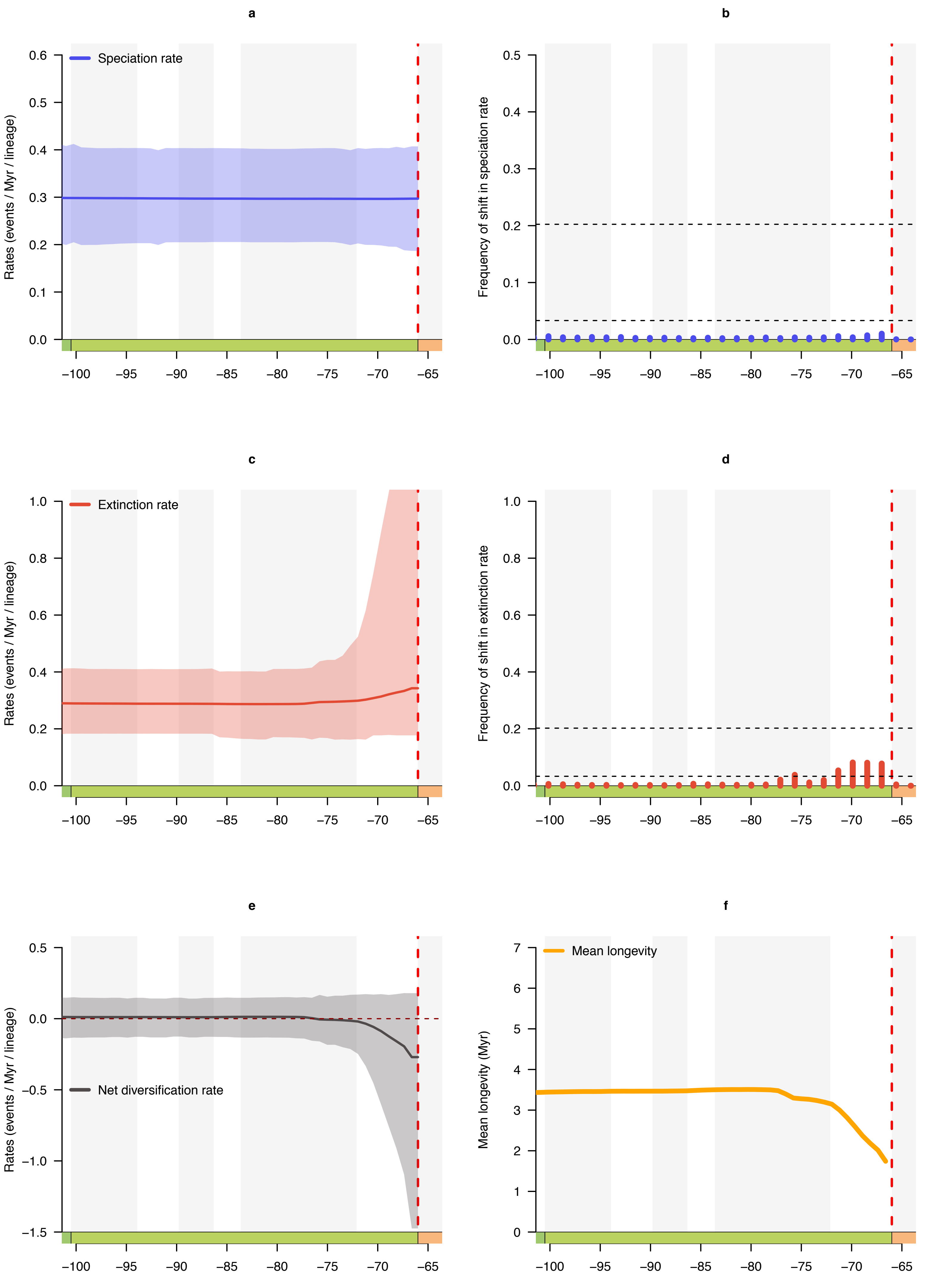
a
Speciation rate
Rates (events / Myr / lineage)
b
Frequency of shift in speciation rate
c
Extinction rate
Rates (events / Myr / lineage)
d
Frequency of shift in extinction rate
e
Net diversification rate
Rates (events / Myr / lineage)
f
Mean longevity
Mean longevity (Myr)
−100
−95
−90
−85
−80
−75
−70
−65

Supplementary Figure 8. Diversification and diversity dynamics of Late Cretaceous hadrosaurs. The Bayesian RJMCMC model estimates speciation rate (**a**), time shifts in speciation rate (**b**), extinction rate (**c**), time shifts in extinction rate (**d**), net diversification rate (**e**, speciation minus extinction) rates, and species longevity in million years (**f**). Solid lines indicate mean posterior rates and shaded areas show 95% CI. As in the BDMCMC model, net diversification decreased and became negative ~70 Myrs ago (middle Maastrichtian), mostly due to an decrease of speciation rate. The vertical dashed red line indicates the Cretaceous-Palaeogene mass extinction (66 Myrs ago).

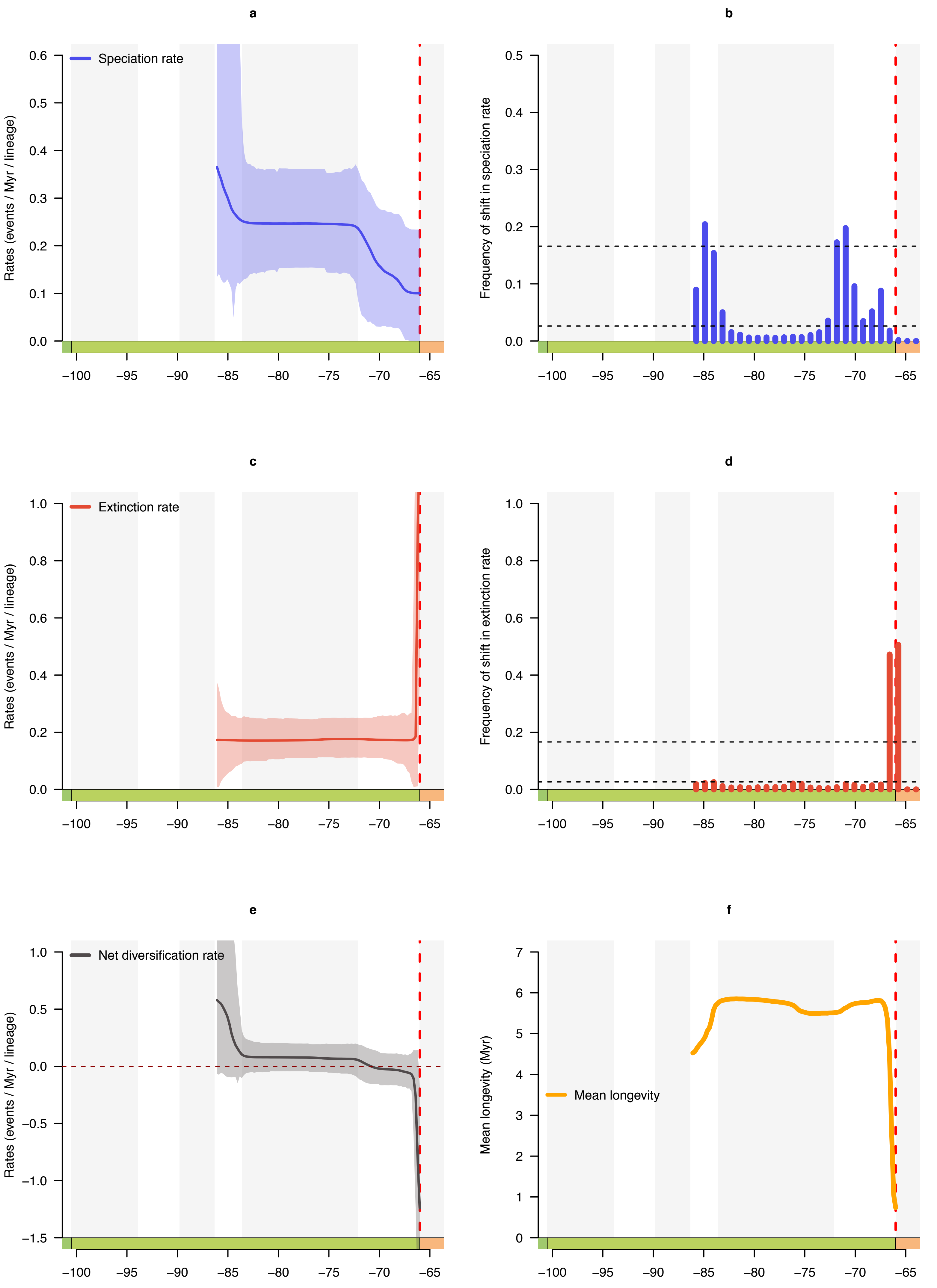

a
Speciation rate
Rates (events / Myr / lineage)
b
Frequency of shift in speciation rate
c
Extinction rate
Rates (events / Myr / lineage)
d
Frequency of shift in extinction rate
e
Net diversification rate
Rates (events / Myr / lineage)
f
Mean longevity
Mean longevity (Myr)
−100
−95
−90
−85
−80
−75
−70
−65

Supplementary Figure 9. Diversification and diversity dynamics of Late Cretaceous troodontids. The Bayesian RJMCMC model estimates speciation rate (**a**), time shifts in speciation rate (**b**), extinction rate (**c**), time shifts in extinction rate (**d**), net diversification rate (**e**, speciation minus extinction) rates, and species longevity in million years (**f**). Solid lines indicate mean posterior rates and shaded areas show 95% CI. As in the BDMCMC model, net diversification remained quite constant through time, although it became negative ~72 Myrs ago (early Maastrichtian) due to a slight decrease of speciation rate. The vertical dashed red line indicates the Cretaceous-Palaeogene mass extinction (66 Myrs ago).

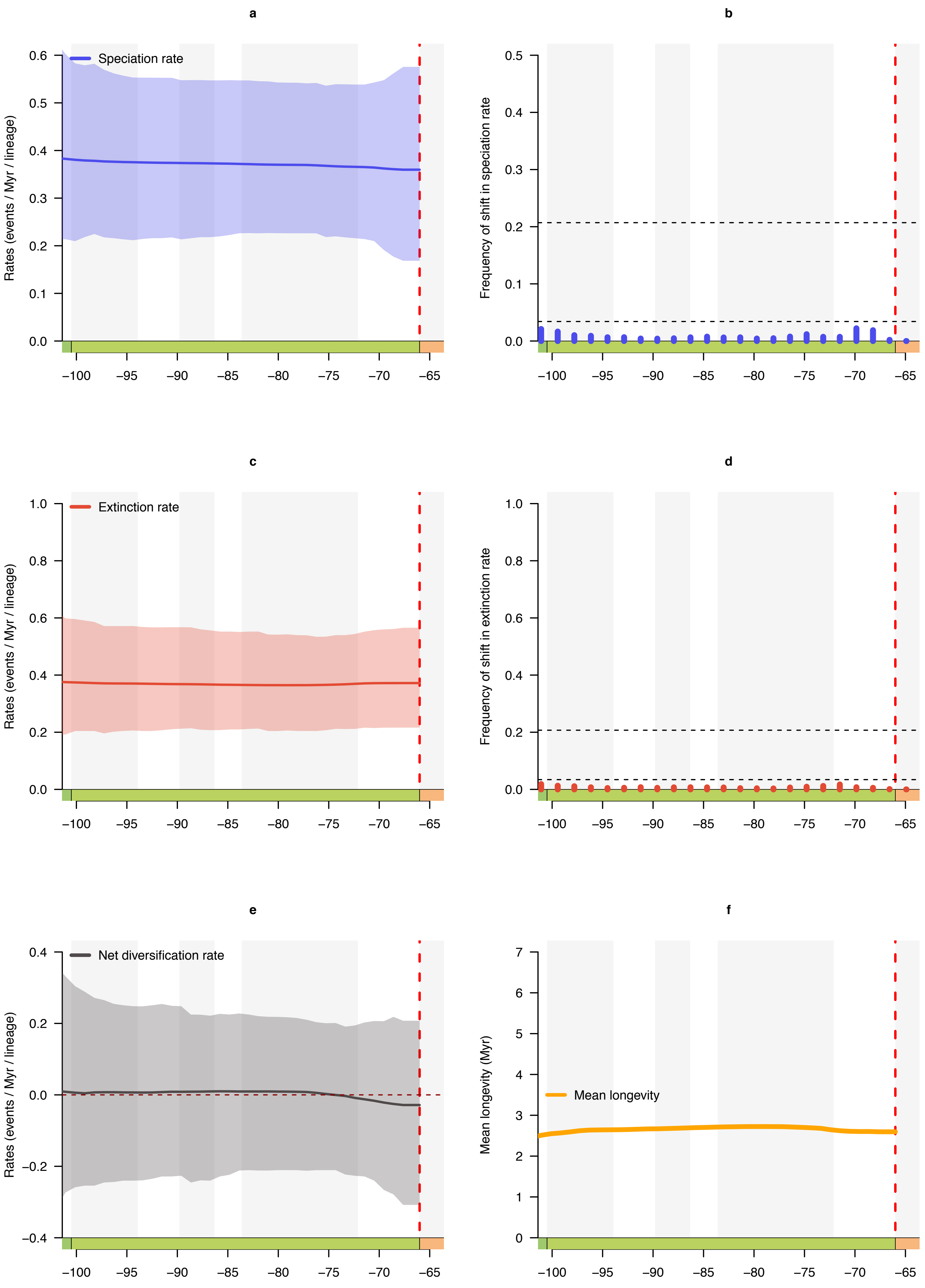

a
Speciation rate
Rates (events / Myr / lineage)
b
Frequency of shift in speciation rate
c
Extinction rate
Rates (events / Myr / lineage)
d
Frequency of shift in extinction rate
e
Net diversification rate
Rates (events / Myr / lineage)
f
Mean longevity
Mean longevity (Myr)
−100
−95
−90
−85
−80
−75
−70
−65

Supplementary Figure 10. Diversification and diversity dynamics of Late Cretaceous tyrannosaurs. The Bayesian RJMCMC model estimates speciation rate (**a**), time shifts in speciation rate (**b**), extinction rate (**c**), time shifts in extinction rate (**d**), net diversification rate (**e**, speciation minus extinction) rates, and species longevity in million years (**f**). Solid lines indicate mean posterior rates and shaded areas show 95% CI. As in the BDMCMC model, net diversification decrease and became negative ~72 Myrs ago (early Maastrichtian) due to a decrease of speciation rate. The vertical dashed red line indicates the Cretaceous-Palaeogene mass extinction (66 Myrs ago).

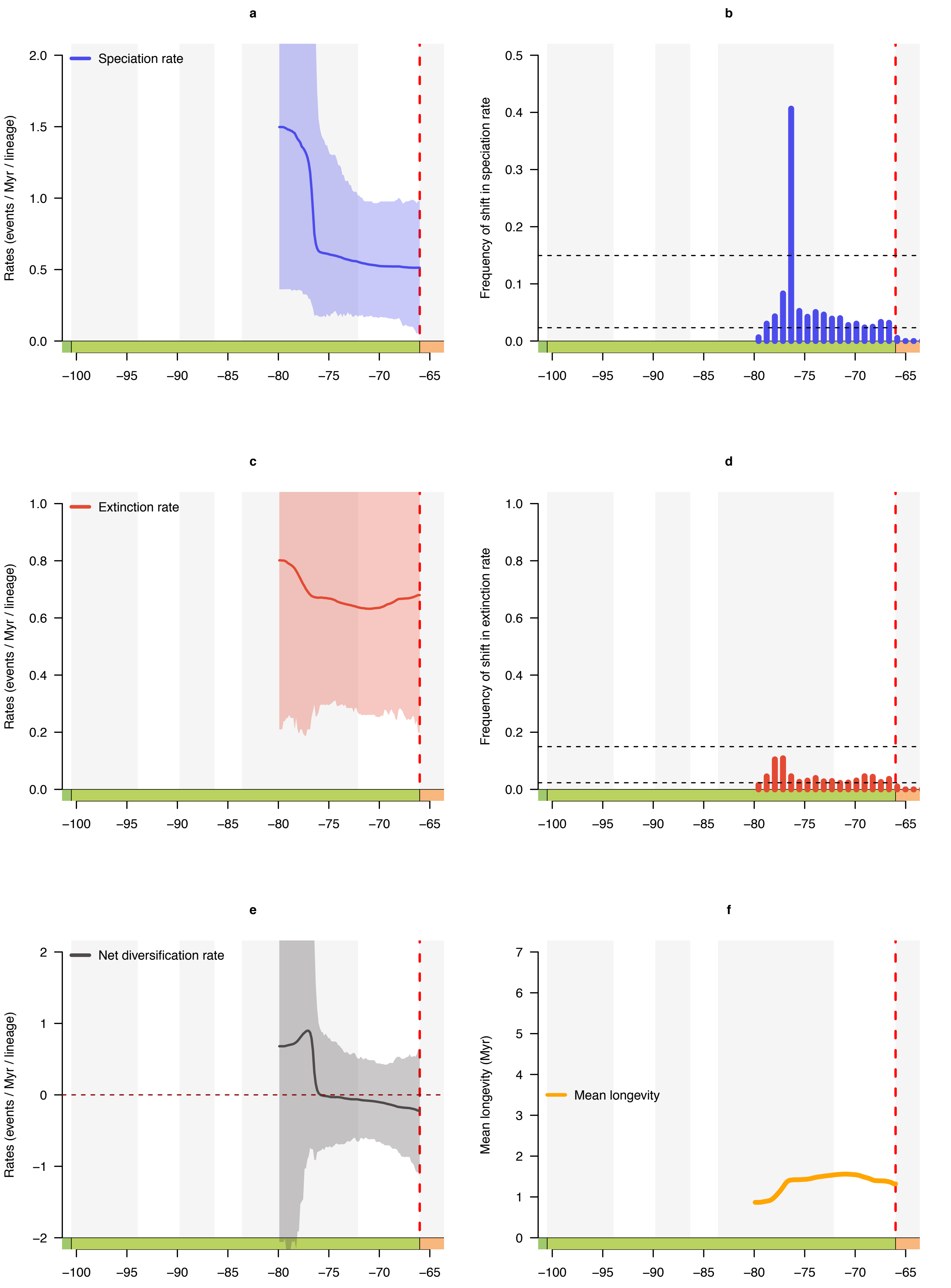
a
b
c
d
e
f
Speciation rate
Extinction rate
Net diversification rate
Mean longevity
Rates (events / Myr / lineage)
Frequency of shift in speciation rate
Frequency of shift in extinction rate
Mean longevity (Myr)
−100
−95
−90
−85
−80
−75
−70
−65

Supplementary Figure 11. Graphical visualizations of the seven paleo-environmental variables tested as abiotic or biotic drivers of dinosaur diversification. The variations of global deep-sea temperatures (**a**) and the fluctuations of sea level (**b**) are estimated from relative proportions of different oxygen isotopes ($\delta^{18}O$) in samples of benthic foraminifer shells. The continental fragmentation (**c**) was estimated using paleogeographic reconstructions for 1-million-year time intervals. The relative diversity of angiosperms (**d**), gymnosperms (**e**), non-Polypodiales ferns (**f**), and Polypodiales ferns (**g**) are estimated from analyses of the vascular plant fossil record (see *Methods*). The vertical dashed black line indicates the Cretaceous-Palaeogene mass extinction (66 Myrs ago).

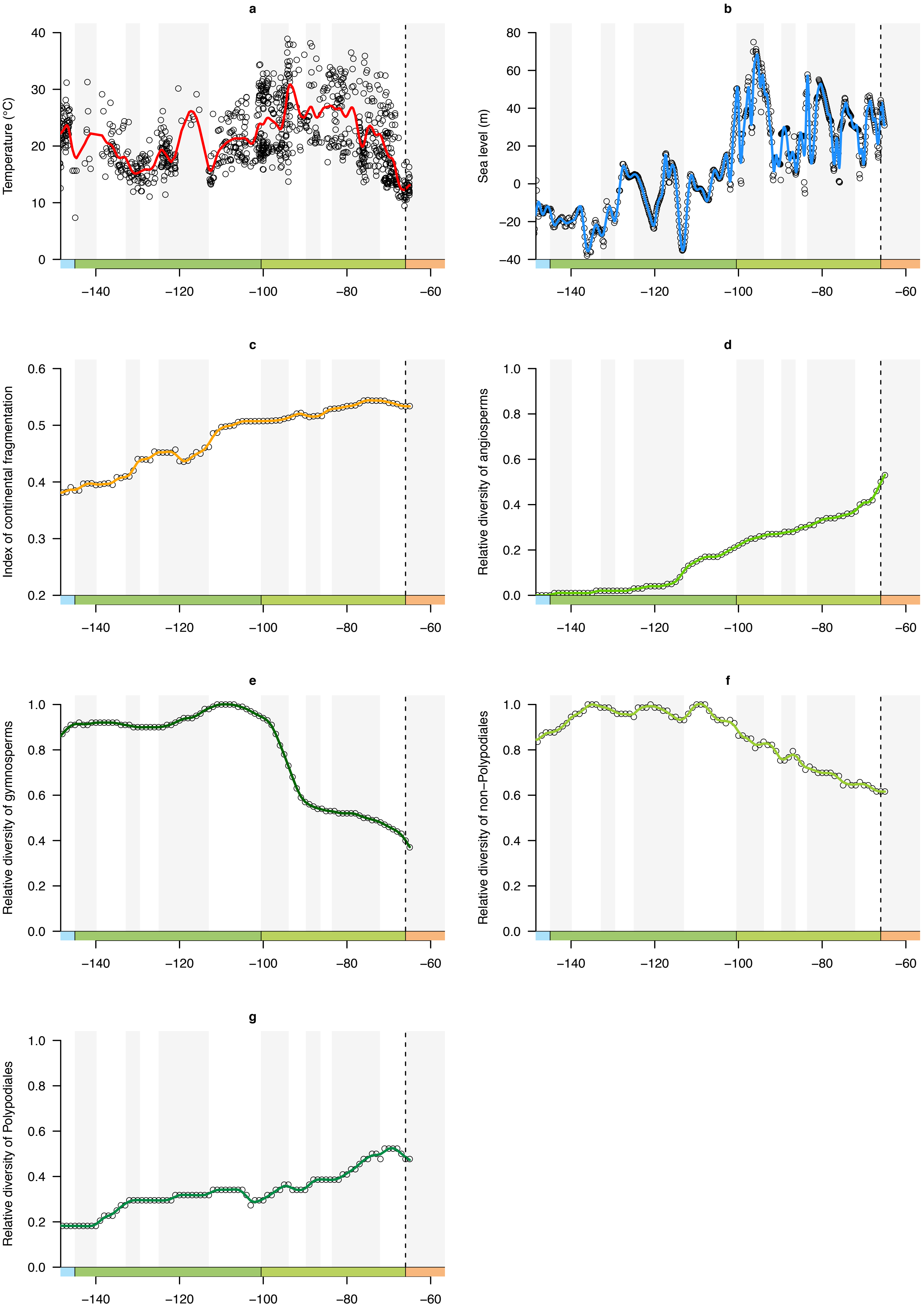

a
Temperature (°C)
b
Sea level (m)
c
Index of continental fragmentation
d
Relative diversity of angiosperms
e
Relative diversity of gymnosperms
f
Relative diversity of non−Polypodiales
g
Relative diversity of Polypodiales
−140
−120
−100
−80
−60

Supplementary Figure 12. Diversity trajectories and the effect of competition on diversification rates of North American dinosaur families. The diversity trajectories of the three carnivorous and three herbivorous dinosaur families are estimated at the North American scale (**a**). Reconstructions of diversity trajectories are the mean of 10 replicates, incorporating uncertainties around the age of the fossil occurrences. The network (**b**) shows the diversity-dependent effects within and between clades on speciation and extinction rates (only significant correlations are shown, Supplementary Table 3). Each arrow indicates the type of interaction imposed by a given group over another, which quantifies the proportion of rate change (decrease/increase for speciation or extinction) associated with the addition of one species of the competing group. Analyses for the global dinosaurs are presented in Fig. 5 and Supplementary Table 2. Abbreviations: Apt: Aptian, Alb: Albian, Cen: Cenomanian, Tur: Turonian, Con: Coniacian, Sant: Santonian, Cam: Campanian, Maa: Maastrichtian, Dan: Danian, Pg: Paleogene, and K-Pg: Cretaceous-Palaeogene mass extinction (66 Myrs ago). Dinosaur pictures courtesy of Fred Wierum (© Wikimedia Commons), Debivort (© Wikimedia Commons), Jack Mayer Wood (© Wikimedia Commons): https://creativecommons.org/licenses/by-sa/4.0/. Palaeomap used with permission © 2020 Colorado Plateau Geosystems Inc. Asteroid icon made by Fabien Condamine.

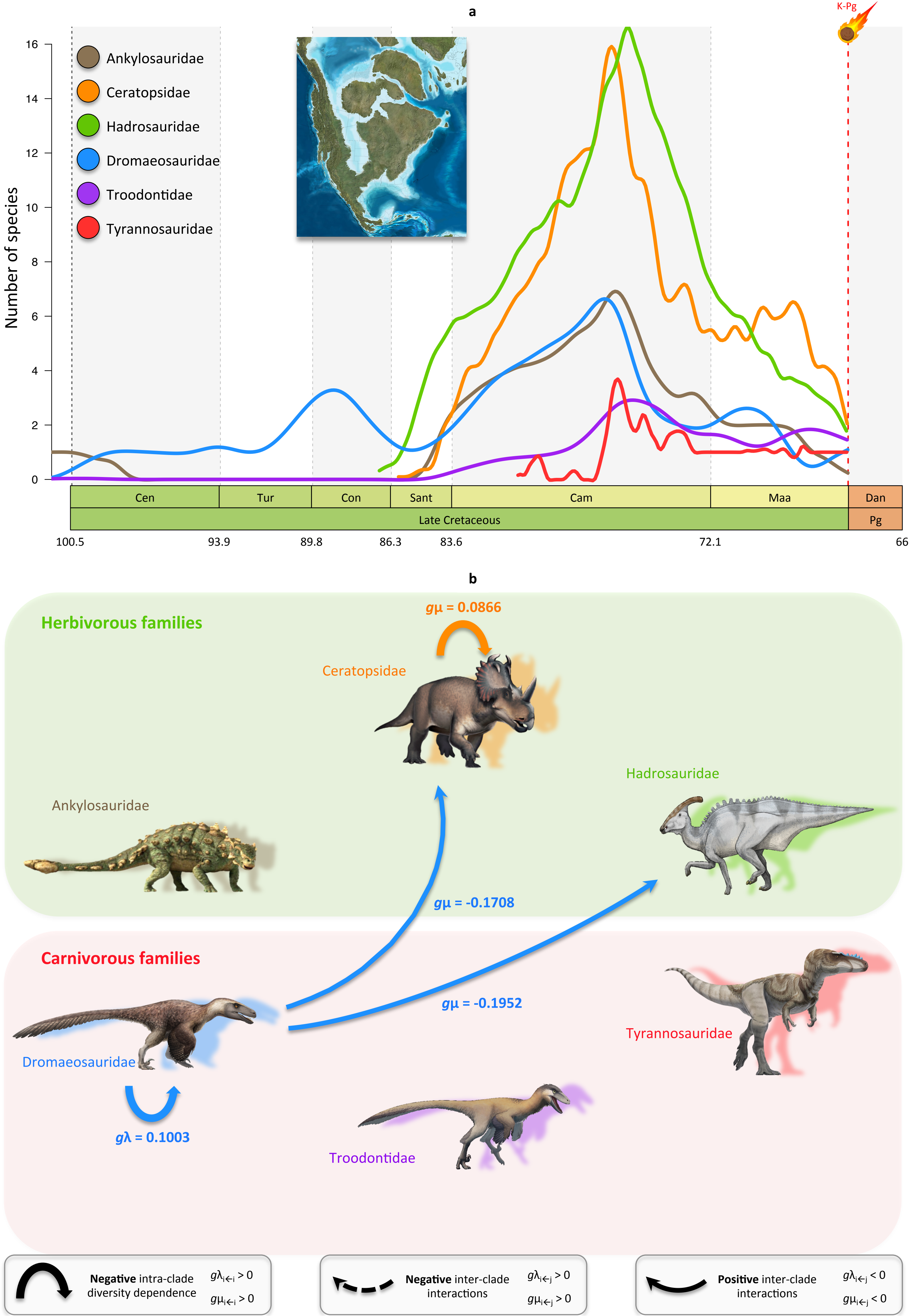

a
K-Pg
Number of species
Ankylosauridae
Ceratopsidae
Hadrosauridae
Dromaeosauridae
Troodontidae
Tyrannosauridae
Cen
Tur
Con
Sant
Cam
Maa
Dan
Late Cretaceous
Pg
100.5
93.9
89.8
86.3
83.6
72.1
66
b
Herbivorous families
Carnivorous families
gμ = 0.0866
gμ = -0.1708
gμ = -0.1952
gλ = 0.1003
Negative intra-clade diversity dependence
Negative inter-clade interactions
Positive inter-clade interactions

Supplementary Table 1. Posterior parameter estimates for the MBD model applied to all families. Baseline speciation and extinction rates (λ0 and μ0) and correlation parameters (*G*λ and *G*μ). The environmental drivers are numbered as follows: (0) diversity of all dinosaurs through time, (1) diversity of carnivorous dinosaurs through time, (2) diversity of herbivorous dinosaurs through time, (3) angiosperm diversity through time, (4) gymnosperm diversity through time, (5) non-Polypodiales ferns diversity through time, (6) Polypodiales ferns diversity through time, (7) continental fragmentation through time, (8) sea-level fluctuations through time, and (9) global temperature changes through time. Shrinkage weights (ω), based on local and global shrinkage parameters, and global shrinkage (τ). Shrinkage weights greater than 0.5 (highlighted in bold) indicate significant evidence for correlation (positive or negative depending on the respective *G*λ or *G*μ value).

Parameters		Exponential model		Linear model	
		Median	95% HPD	Median	95% HPD
Baseline rates	λ0	0.46	[0.0104, 1.1167]	0.1782	[0.0194, 0.5703]
	μ0	0.6727	[0.194, 1.3075]	0.5817	[0.2128, 1.0027]
Correlation parameters to speciation	*G*λ0_0	1.11E-03	[-0.0126, 0.0178]	6.00E-04	[-0.0222, 0.0437]
	*G*λ0_1	0.0162	[-0.0147, 0.0649]	0.0134	[-0.0388, 0.2111]
	*G*λ0_2	3.03E-04	[-0.0165, 0.0199]	3.62E-04	[-0.0388, 0.0438]
	*G*λ0_3	-1.064	[-5.3388, 1.174]	-0.0933	[-4.1822, 2.4822]
	*G*λ0_4	0.2208	[-1.5506, 2.1899]	0.3348	[-1.2438, 24.1626]
	*G*λ0_5	-0.1227	[-4.5713, 5.6763]	0.0625	[-4.9594, 8.3138]
	*G*λ0_6	0.6965	[-2.9456, 8.0272]	0.0932	[-3.4696, 7.672]
	*G*λ0_7	0.5471	[-5.0261, 7.2582]	0.2041	[-7.2036, 23.459]
	*G*λ0_8	-0.0023	[-0.0115, 3.6005E-3]	-0.0004	[-0.0156, 0.011]
	*G*λ0_9	0.0109	[-0.0261, 0.0682]	6.49E-03	[-0.0518, 0.1751]
Correlation parameters to extinction	*G*μ0_0	-0.0036	[-0.0584, 0.0391]	-0.0009	[-0.0309, 0.0181]
	*G*μ0_1	0.0468	[-0.0097, 0.1215]	0.0218	[-0.0103, 0.0791]
	***G*μ0_2**	**-0.0353**	**[-0.0862, 0]**	**-0.0155**	**[-0.0479, 0]**
	*G*μ0_3	0.663	[-0.9261, 3.3388]	0.0627	[-1.2755, 2.264]
	*G*μ0_4	-0.2726	[-1.914, 0.8805]	-0.0353	[-0.8992, 0.8072]
	*G*μ0_5	-0.9501	[-3.9205, 1.0223]	-0.5712	[-2.3039, 0.8612]
	*G*μ0_6	0.6882	[-1.4416, 4.2949]	0.3454	[-1.137, 5.5999]
	*G*μ0_7	0.054	[-5.5823, 5.8856]	-0.0585	[-6.2575, 3.9169]
	*G*μ0_8	-0.0012	[-0.0085, 4.6597E-3]	-0.0012	[-0.0074, 2.0571E-3]
	***G*μ0_9**	**-0.0809**	**[-0.132, -0.028]**	**-0.0445**	**[-0.0944, -0.0039]**
Shrinkage weights (speciation)	ωλ0_0	0.186	[2.0382E-9, 0.7599]	0.0721	[3.6138E-10, 0.8931]
	ωλ0_1	0.2076	[7.8624E-10, 0.7742]	0.1368	[4.2534E-8, 0.9427]
	ωλ0_2	0.1689	[9.4718E-11, 0.7107]	0.0646	[1.2187E-11, 0.8968]
	ωλ0_3	0.3051	[4.0713E-10, 0.9253]	0.112	[1.0145E-8, 0.9089]
	ωλ0_4	0.2208	[5.1987E-11, 0.8163]	0.1647	[3.7309E-8, 0.9943]

	ωλ0_5	0.2646	[1.0942E-7, 0.8958]	0.0969	[5.5201E-10, 0.9467]
	ωλ0_6	0.2544	[6.7014E-9, 0.9183]	0.0881	[2.9343E-9, 0.9484]
	ωλ0_7	0.2058	[7.4694E-9, 0.8043]	0.0783	[6.6339E-9, 0.9622]
	ωλ0_8	0.2223	[7.6905E-11, 0.8105]	0.0871	[1.0617E-7, 0.8637]
	ωλ0_9	0.164	[5.7246E-8, 0.7308]	0.0656	[3.369E-9, 0.8867]
Shrinkage weights (extinction)	ωμ0_0	0.3354	[4.5112E-8, 0.9423]	0.1186	[2.5856E-9, 0.8525]
	ωμ0_1	0.3902	[5.3377E-8, 0.9183]	0.1584	[2.1004E-9, 0.8547]
	**ωμ0_2**	**0.5554**	**[3.5436E-6, 0.9721]**	0.2898	[1.3866E-7, 0.9128]
	ωμ0_3	0.2604	[2.69E-10, 0.8588]	0.0644	[6.4672E-9, 0.7983]
	ωμ0_4	0.2098	[2.2316E-9, 0.797]	0.0489	[1.581E-10, 0.6827]
	ωμ0_5	0.2595	[8.2911E-9, 0.8474]	0.1061	[1.0711E-8, 0.7682]
	ωμ0_6	0.2434	[6.0399E-8, 0.8569]	0.0999	[2.6247E-8, 0.9036]
	ωμ0_7	0.2012	[8.0198E-9, 0.7962]	0.054	[7.9448E-12, 0.8023]
	ωμ0_8	0.1837	[8.1776E-9, 0.7439]	0.069	[1.9047E-10, 0.7439]
	**ωμ0_9**	**0.5061**	**[0.0979, 0.978]**	0.2713	[1.6412E-5, 0.8765]
Global shrinkage	τ	0.4131	[0.085, 0.9571]	0.3546	[0.0446, 1.1134]
Hyperprior	η	4.0051	[2.4316, 7.0632]	4.1757	[2.1167, 5.8446]

Supplementary Table 2. Posterior parameter estimates for the MCDD model applied to all families. The clades are numbered as follows: (0) Ankylosauridae, (1) Ceratopsidae, (2) Dromaeosauridae, (3) Hadrosauridae, (4) Troodontidae, and (5) Tyrannosauridae. Baseline speciation represents the speciation rate for a clade in absence of diversity dependence. $g\lambda0\_0$ denotes for diversity dependence in the clade 0. $g\lambda0\_1 > 0$ indicates the diversity of clade 1 negatively correlates with speciation of clade 0 (increasing diversity of clade 1 correlates with low speciation of clade 0). $g\lambda0\_1 < 0$ indicates the diversity of clade 1 positively correlates with speciation of clade 0 (increasing diversity of clade 1 correlates with high speciation of clade 0). Bold values are significant correlations.

**Parameters**		**Exponential model**		**Linear model**	
		**Median**	**95% HPD**	**Median**	**95% HPD**
Baseline speciation	$\lambda0$	0.2286	[0.0852, 0.4822]	0.2296	[0.0838, 0.4806]
	$\lambda1$	0.3589	[0.0726, 0.7502]	0.3539	[0.0699, 0.7354]
	$\lambda2$	0.4215	[0.2082, 0.6659]	0.4192	[0.2077, 0.6636]
	$\lambda3$	0.3535	[0.1007, 0.6564]	0.3505	[0.0976, 0.6481]
	$\lambda4$	0.5214	[0.2687, 0.8262]	0.52	[0.2673, 0.8258]
	$\lambda5$	0.7639	[0.0835, 2.3089]	0.7454	[0.0785, 2.2976]
Baseline extinction	$\mu0$	0.1218	[0.0443, 0.2333]	0.1227	[0.0446, 0.235]
	$\mu1$	0.1925	[0.0475, 0.4971]	0.1956	[0.0506, 0.5041]
	$\mu2$	0.275	[0.138, 0.4456]	0.2739	[0.1388, 0.4442]
	$\mu3$	0.2616	[0.0784, 0.4906]	0.2604	[0.0843, 0.4919]
	$\mu4$	0.4172	[0.208, 0.686]	0.417	[0.2036, 0.675]
	$\mu5$	0.4181	[0.0646, 1.1572]	0.4179	[0.0667, 1.154]
Intra-family diversity dependence	$g\lambda0\_0$	2.76E-03	[-0.0298, 0.2993]	0.0105	[-0.0268, 0.2999]
	$g\mu0\_0$	0	[-0.0681, 0.291]	0	[-0.0742, 0.2911]
	$g\lambda1\_1$	**0.1308**	**[0, 0.2756]**	**0.1286**	**[0, 0.276]**
	$g\mu1\_1$	0	[-0.1506, 0.1889]	0	[-0.1588, 0.1848]
	$g\lambda2\_2$	**0.1661**	**[0, 0.2879]**	**0.1654**	**[0, 0.2878]**
	$g\mu2\_2$	**0.1688**	**[0, 0.292]**	**0.1695**	**[0, 0.2915]**
	$g\lambda3\_3$	**0.0714**	**[0, 0.1073]**	**0.0712**	**[0, 0.1075]**
	$g\mu3\_3$	0	[-0.0071, 0.1956]	0	[-0.0072, 0.1918]
	$g\lambda4\_4$	**0.1836**	**[0, 0.2909]**	**0.1812**	**[0, 0.2908]**
	$g\mu4\_4$	**0.0905**	**[0, 0.2944]**	**0.0899**	**[0, 0.2949]**
	$g\lambda5\_5$	**0.2342**	**[0, 0.2991]**	**0.2322**	**[0, 0.2991]**
	$g\mu5\_5$	0	[-0.1456, 0.3]	0	[-0.1504, 0.3]
Competition or positive interaction over ankylosaurid speciation	$g\lambda0\_1$	-0.0525	[-0.2029, 3.7289E-3]	-0.0525	[-0.2055, 4.807E-4]
	$g\lambda0\_2$	0	[-0.0868, 0.2804]	0	[-0.0949, 0.277]
	$g\lambda0\_3$	0	[-0.1006, 0.0428]	0	[-0.0992, 0.0425]
	$g\lambda0\_4$	0	[-0.1567, 0.1951]	0	[-0.1526, 0.1967]
	$g\lambda0\_5$	0	[-0.2944, 0.1794]	0	[-0.2992, 0.175]

Competition or positive interaction over ankylosaurid extinction	*gμ0_1*	0	[-0.3, 0.0569]	0	[-0.3, 0.0577]
	gμ0_2	0	[-0.0929, 0.2998]	0	[-0.0923, 0.2998]
	gμ0_3	**5.51E-03**	**[0, 0.2616]**	0	[-0.0001, 0.2583]
	gμ0_4	0	[-0.2125, 0.3]	0	[-0.2065, 0.3]
	gμ0_5	0	[-0.1286, 0.2999]	0	[-0.1339, 0.3]
Competition or positive interaction over ceratopsid speciation	*gλ1_0*	**-0.1185**	**[-0.2983, 0]**	**-0.1147**	**[-0.2984, 0]**
	gλ1_2	0	[-0.2999, 0.0225]	0	[-0.2998, 0.0212]
	gλ1_3	0	[-0.1856, 0.0543]	0	[-0.1924, 0.0447]
	gλ1_4	0	[-0.2981, 0.1341]	0	[-0.2985, 0.1303]
	gλ1_5	0	[-0.1068, 0.3]	0	[-0.109, 0.3]
Competition or positive interaction over ceratopsid extinction	*gμ1_0*	0	[-0.3, 0.0526]	0	[-0.3, 0.0559]
	gμ1_2	0	[-0.2999, 0.1488]	0	[-0.3, 0.1408]
	gμ1_3	**0.0906**	**[0, 0.2859]**	**0.0893**	**[0, 0.2857]**
	gμ1_4	0.1446	[-0.0061, 0.3]	0.1456	[-0.0113, 0.3]
	gμ1_5	0	[-0.1577, 0.3]	0	[-0.161, 0.3]
Competition or positive interaction over dromaeosaurid speciation	*gλ2_0*	0	[-0.2237, 0.0171]	0	[-0.2255, 0.0149]
	gλ2_1	0	[-0.0452, 0.0811]	0	[-0.0501, 0.0762]
	gλ2_3	0	[-0.0706, 2.1077E-3]	0	[-0.0704, 2.1922E-3]
	gλ2_4	0	[-0.2878, 2.3976E-4]	-0.0002	[-0.2883, 4.5311E-7]
	gλ2_5	0	[-0.296, 0.146]	0	[-0.2987, 0.1392]
Competition or positive interaction over dromaeosaurid extinction	*gμ2_0*	**-0.1656**	**[-0.2865, 0]**	**-0.1668**	**[-0.2864, 0]**
	gμ2_1	0	[-0.2313, 0.0794]	0	[-0.2299, 0.081]
	gμ2_3	0	[-0.0131, 0.1249]	0	[-0.0159, 0.1232]
	gμ2_4	0	[-0.1724, 0.2572]	0	[-0.1636, 0.2631]
	gμ2_5	3.12E-03	[-0.0645, 0.3]	5.99E-03	[-0.0578, 0.3]
Competition or positive interaction over hadrosaurid speciation	*gλ3_0*	**-0.0491**	**[-0.2807, 0]**	**-0.0505**	**[-0.2796, 0]**
	gλ3_1	0	[-0.0779, 0.1127]	0	[-0.0768, 0.114]
	gλ3_2	0	[-0.1857, 0.0674]	0	[-0.1798, 0.078]
	gλ3_4	-0.0899	[-0.2554, 6.3557E-4]	-0.0894	[-0.2564, 1.0415E-5]
	gλ3_5	0	[-0.264, 0.2035]	0	[-0.2694, 0.1934]
Competition or positive interaction over hadrosaurid extinction	*gμ3_0*	0	[-0.1707, 0.1498]	0	[-0.1807, 0.1416]
	gμ3_1	0	[-0.2257, 0.0139]	0	[-0.2124, 0.0231]
	gμ3_2	**-0.0939**	**[-0.2879, 0]**	**-0.0961**	**[-0.2876, 0]**
	gμ3_4	0	[-0.065, 0.2484]	0	[-0.0551, 0.2624]
	gμ3_5	0	[-0.2756, 0.0958]	0	[-0.2935, 0.0752]
Competition or positive interaction over troodontid speciation	*gλ4_0*	0	[-0.2681, 0.1077]	0	[-0.2741, 0.1015]
	gλ4_1	0	[-0.1474, 0.0768]	0	[-0.1477, 0.0776]
	gλ4_2	0	[-0.2805, 0.037]	0	[-0.2797, 0.0359]
	gλ4_3	0	[-0.0746, 0.0309]	0	[-0.0707, 0.035]
	gλ4_5	0	[-0.2991, 0.1623]	0	[-0.286, 0.179]
Competition or	*gμ4_0*	0	[-0.2721, 0.0353]	0	[-0.2724, 0.0419]

positive interaction over troodontid extinction	*gμ4_1*	0	[-0.2118, 0.0645]	0	[-0.2235, 0.0625]
	gμ4_2	0	[-0.0745, 0.2993]	0	[-0.0796, 0.2997]
	gμ4_3	0	[-0.0348, 0.0703]	0	[-0.0393, 0.0753]
	gμ4_5	0	[-0.1584, 0.3]	0	[-0.1627, 0.3]
Competition or positive interaction over tyrannosaurid speciation	*gλ5_0*	0	[-0.2998, 6.7882E-3]	0	[-0.2999, 6.2419E-3]
	gλ5_1	0	[-0.2988, 2.9988E-3]	0	[-0.2999, 3.8812E-4]
	gλ5_2	0	[-0.2997, 0.0999]	0	[-0.3, 0.112]
	gλ5_3	0	[-0.0745, 0.0986]	0	[-0.088, 0.0982]
	gλ5_4	0	[-0.131, 0.2712]	0	[-0.1249, 0.2805]
Competition or positive interaction over tyrannosaurid extinction	*gμ5_0*	0	[-0.0814, 0.2998]	0	[-0.078, 0.3]
	gμ5_1	0	[-0.0583, 0.3]	0	[-0.0601, 0.3]
	gμ5_2	0	[-0.2969, 0.189]	0	[-0.2931, 0.196]
	gμ5_3	0	[-0.0507, 0.2553]	0	[-0.0537, 0.2537]
	gμ5_4	0	[-0.2998, 0.1611]	0	[-0.2999, 0.158]
Hyperprior	η0	0.5427	[0.2067, 0.8811]	0.5438	[0.2008, 0.876]
	η1	0.4807	[0.15, 0.813]	0.482	[0.1566, 0.8154]
	η2	0.5088	[0.1907, 0.8339]	0.5076	[0.1849, 0.8305]
	η3	0.506	[0.181, 0.8213]	0.5052	[0.1818, 0.8215]
	η4	0.557	[0.2221, 0.8751]	0.5572	[0.2271, 0.8826]
	η5	0.5667	[0.2185, 0.8896]	0.5632	[0.2234, 0.894]

Supplementary Table 3. Posterior parameter estimates for the MCDD model applied to the North American species. The clades are numbered as follows: (0) Ankylosauridae, (1) Ceratopsidae, (2) Dromaeosauridae, (3) Hadrosauridae, (4) Troodontidae, and (5) Tyrannosauridae. Baseline speciation represents the speciation rate for a clade in absence of diversity dependence. $g\lambda0\_0$ denotes for diversity dependence in the clade 0. $g\lambda0\_1 > 0$ indicates the diversity of clade 1 negatively correlates with speciation of clade 0 (increasing diversity of clade 1 correlates with low speciation of clade 0). $g\lambda0\_1 < 0$ indicates the diversity of clade 1 positively correlates with speciation of clade 0 (increasing diversity of clade 1 correlates with high speciation of clade 0). Bold values are significant correlations.

**Parameters**		**Exponential model**		**Linear model**	
		**Median**	**95% HPD**	**Median**	**95% HPD**
Baseline speciation	λ0	0.1925	[0.0432, 0.4264]	0.1919	[0.0438, 0.4256]
	λ1	0.4307	[0.1552, 0.7722]	0.4318	[0.1561, 0.7795]
	λ2	0.312	[0.141, 0.5283]	0.3122	[0.1403, 0.5284]
	λ3	0.2248	[0.0818, 0.3916]	0.224	[0.0805, 0.3917]
	λ4	0.3805	[0.0936, 0.8353]	0.3783	[0.0895, 0.8274]
	λ5	0.3621	[0.0803, 0.9824]	0.3615	[0.0826, 0.9799]
Baseline extinction	μ0	0.2017	[0.0453, 0.4343]	0.2035	[0.0425, 0.4371]
	μ1	0.3766	[0.1638, 0.63]	0.3745	[0.1641, 0.6274]
	μ2	0.244	[0.1012, 0.4273]	0.2444	[0.1011, 0.4255]
	μ3	0.3105	[0.0878, 0.5649]	0.3103	[0.0862, 0.5619]
	μ4	0.4486	[0.1187, 0.9622]	0.4468	[0.1246, 0.9612]
	μ5	0.3854	[0.0932, 0.9066]	0.3852	[0.1001, 0.9066]
Intra-family diversity dependence	*gλ0_0*	0	[-0.0871, 0.3]	0	[-0.0913, 0.3]
	gμ0_0	0	[-0.1747, 0.3]	0	[-0.1772, 0.3]
	gλ1_1	0.0275	[-0.0006, 0.2444]	0.0289	[-0.0012, 0.2454]
	gμ1_1	**0.0866**	**[0, 0.2884]**	**0.085**	**[0, 0.2899]**
	gλ2_2	**0.1003**	**[0, 0.2851]**	**0.0997**	**[0, 0.2863]**
	gμ2_2	0	[-0.173, 0.2174]	0	[-0.1703, 0.2182]
	gλ3_3	0	[-0.0627, 0.1475]	0	[-0.0636, 0.1487]
	gμ3_3	0	[-0.0032, 0.2505]	0	[-0.0043, 0.2469]
	gλ4_4	0.1355	[-0.0455, 0.3]	0.1339	[-0.044, 0.3]
	gμ4_4	0.0815	[-0.0236, 0.3]	0.0808	[-0.0236, 0.3]
	gλ5_5	0	[-0.1241, 0.3]	0	[-0.1215, 0.3]
	gμ5_5	0	[-0.1666, 0.3]	0	[-0.167, 0.3]
Competition or positive interaction over ankylosaurid speciation	*gλ0_1*	0	[-0.2058, 0.1962]	0	[-0.2015, 0.2045]
	gλ0_2	0	[-0.2999, 0.1546]	0	[-0.3, 0.1493]
	gλ0_3	0	[-0.2932, 2.2692E-5]	0	[-0.2949, 2.9096E-6]
	gλ0_4	0	[-0.2268, 0.2993]	0	[-0.2254, 0.2996]
	gλ0_5	0	[-0.2388, 0.2799]	0	[-0.2463, 0.272]

Competition or positive interaction over ankylosaurid extinction	*gμ0_1*	0	[-0.21, 0.2359]	0	[-0.212, 0.2331]
	gμ0_2	-0.1256	[-0.3, 0.0327]	-0.1272	[-0.3, 0.0326]
	gμ0_3	0	[-0.04, 0.2749]	0	[-0.029, 0.2885]
	gμ0_4	2.03E-03	[-0.0948, 0.3]	5.59E-03	[-0.0877, 0.3]
	gμ0_5	0	[-0.0945, 0.3]	0	[-0.093, 0.3]
Competition or positive interaction over ceratopsid speciation	*gλ1_0*	0	[-0.3, 0.1105]	**0**	**[-0.3, 0.1038]**
	gλ1_2	0	[-0.2986, 9.7837E-3]	0	[-0.2901, 0.02]
	gλ1_3	0	[-0.2003, 0.0441]	0	[-0.2087, 0.0401]
	gλ1_4	0	[-0.179, 0.2998]	0	[-0.1816, 0.2971]
	gλ1_5	0	[-0.0922, 0.3]	0	[-0.0883, 0.3]
Competition or positive interaction over ceratopsid extinction	*gμ1_0*	-0.0673	[-0.3, 0.1025]	-0.0637	[-0.3, 0.1124]
	gμ1_2	**-0.1708**	**[-0.2981, 0]**	**-0.1746**	**[-0.2985, 0]**
	gμ1_3	0	[-0.2371, 0.112]	0	[-0.2333, 0.119]
	gμ1_4	0.0372	[-0.0929, 0.3]	0.0447	[-0.0968, 0.3]
	gμ1_5	0	[-0.1378, 0.3]	0	[-0.1359, 0.3]
Competition or positive interaction over dromaeosaurid speciation	*gλ2_0*	0	[-0.2153, 0.2033]	0	[-0.2179, 0.1982]
	gλ2_1	0	[-0.1259, 0.073]	0	[-0.1198, 0.0747]
	gλ2_3	0	[-0.0938, 0.0808]	0	[-0.0898, 0.0828]
	gλ2_4	0	[-0.2999, 0.1827]	0	[-0.3, 0.1811]
	gλ2_5	0	[-0.1621, 0.3]	0	[-0.1626, 0.2999]
Competition or positive interaction over dromaeosaurid extinction	*gμ2_0*	0	[-0.3, 0.0896]	0	[-0.3, 0.0871]
	gμ2_1	0	[-0.1747, 0.1483]	0	[-0.175, 0.1503]
	gμ2_3	0	[-0.1132, 0.15]	0	[-0.1134, 0.1511]
	gμ2_4	0.1296	[-0.0085, 0.3]	0.1337	[-0.0096, 0.3]
	gμ2_5	0.0896	[-0.0451, 0.3]	0.0913	[-0.0455, 0.3]
Competition or positive interaction over hadrosaurid speciation	*gλ3_0*	0	[-0.2946, 0.108]	0	[-0.2991, 0.1045]
	gλ3_1	0	[-0.1172, 0.1315]	0	[-0.1242, 0.1338]
	gλ3_2	0	[-0.2998, 0.0568]	0	[-0.299, 0.0585]
	gλ3_4	0	[-0.2445, 0.2458]	0	[-0.2406, 0.2483]
	gλ3_5	0	[-0.2372, 0.2358]	0	[-0.2485, 0.2249]
Competition or positive interaction over hadrosaurid extinction	*gμ3_0*	0	[-0.3, 0.1165]	0	[-0.3, 0.1161]
	gμ3_1	0	[-0.173, 0.167]	0	[-0.167, 0.1732]
	gμ3_2	**-0.1952**	**[-0.2951, 0]**	**-0.1927**	**[-0.2952, 0]**
	gμ3_4	0	[-0.1747, 0.3]	0	[-0.1702, 0.3]
	gμ3_5	0	[-0.239, 0.2431]	0	[-0.2251, 0.255]
Competition or positive interaction over troodontid speciation	*gλ4_0*	0	[-0.3, 0.1265]	0	[-0.2998, 0.1294]
	gλ4_1	0	[-0.1715, 0.1501]	0	[-0.173, 0.1487]
	gλ4_2	0	[-0.3, 0.0983]	0	[-0.2999, 0.1046]
	gλ4_3	0	[-0.1184, 0.1491]	0	[-0.1198, 0.146]
	gλ4_5	0	[-0.2573, 0.2587]	0	[-0.2526, 0.2637]
Competition or	*gμ4_0*	0	[-0.2998, 0.2317]	0	[-0.2996, 0.2321]

positive interaction over troodontid extinction	*gμ4_1*	0	[-0.2999, 0.0228]	0	[-0.3, 0.0185]
	gμ4_2	0	[-0.3, 0.1432]	0	[-0.3, 0.1412]
	gμ4_3	0	[-0.0849, 0.1997]	0	[-0.0792, 0.2078]
	gμ4_5	0	[-0.1866, 0.3]	0	[-0.1869, 0.3]
Competition or positive interaction over tyrannosaurid speciation	*gλ5_0*	0	[-0.3, 0.1118]	0	[-0.3, 0.1115]
	gλ5_1	0	[-0.3, 0.0148]	0	[-0.3, 0.0158]
	gλ5_2	0	[-0.3, 0.1431]	0	[-0.3, 0.1416]
	gλ5_3	0	[-0.2992, 0.0629]	0	[-0.2999, 0.0585]
	gλ5_4	0	[-0.2196, 0.3]	0	[-0.218, 0.3]
Competition or positive interaction over tyrannosaurid extinction	*gμ5_0*	0	[-0.1509, 0.3]	0	[-0.1544, 0.3]
	gμ5_1	0	[-0.0733, 0.2999]	0	[-0.0677, 0.3]
	gμ5_2	0	[-0.194, 0.2999]	0	[-0.19, 0.2999]
	gμ5_3	0	[-0.0213, 0.2999]	0	[-0.0222, 0.2994]
	gμ5_4	0	[-0.2784, 0.257]	0	[-0.2828, 0.2532]
Hyperprior	η0	0.5004	[0.1674, 0.8383]	0.5001	[0.1594, 0.8333]
	η1	0.4865	[0.1557, 0.8188]	0.4848	[0.151, 0.8163]
	η2	0.5444	[0.2083, 0.8709]	0.5421	[0.2043, 0.8666]
	η3	0.5387	[0.2003, 0.8624]	0.5369	[0.1988, 0.8627]
	η4	0.5367	[0.196, 0.8698]	0.5379	[0.1952, 0.8689]
	η5	0.5064	[0.1749, 0.8394]	0.5053	[0.1707, 0.8344]

Supplementary Table 4. Posterior parameter estimates for the age-dependent extinction (ADE) model for dinosaurs. Preservation rates (*q*) are estimated for the Late Cretaceous entirely, the Campanian and Maastrichtian separately, and the pre-decline and decline phases. Bold values for the shape parameter of the Weibull distribution indicate a significant effect of taxon age on extinction rates. A Weibull shape significantly greater than 1 indicates that extinction probability increases with species age, while a Weibull shape significantly lesser than 1 indicates that extinction rate is higher in younger taxa (Weibull shape not significantly different from 1 means no effect of age on extinction rates).

Late Cretaceous					
**Parameters**	***q***	**alpha**	**Weibull shape**	**Weibull scale**	**Mean longevity (Myrs)**
mean	5.6357	0.3234	1.0796	4.4953	4.3916
median	5.5715	0.3165	1.0774	4.483	4.3759
95% HPD	[4.9067, 6.4723]	[0.2776, 0.3984]	[0.8555, 1.2997]	[3.6713, 5.3735]	[3.6667, 5.1015]

Campanian					
**Parameters**	***q***	**alpha**	**Weibull shape**	**Weibull scale**	**Mean longevity (Myrs)**
mean	4.6985	0.3526	0.8505	3.3063	3.654
median	4.6904	0.35	0.8468	3.2808	3.6097
95% HPD	[4.0541, 5.3331]	[0.2952, 0.4164]	[0.6155, 1.104]	[2.0257, 4.5509]	[2.615, 4.7851]

Maastritchtian					
**Parameters**	***q***	**alpha**	**Weibull shape**	**Weibull scale**	**Mean longevity (Myrs)**
mean	8.88E+00	0.3194	**1.7572**	3.82E+00	3.4295
median	8.8128	0.317	**1.7333**	3.811	3.4107
95% HPD	[7.7415, 10.2212]	[0.2722, 0.3711]	**[1.1362, 2.4768]**	[2.9276, 4.7371]	[2.6608, 4.206]

Pre-decline phase					
**Parameters**	***q***	**alpha**	**Weibull shape**	**Weibull scale**	**Mean longevity (Myrs)**
mean	1.522	0.7981	0.7531	1.4061	1.7263
median	1.4084	0.4834	0.7181	1.2822	1.6017
95% HPD	[0.6424, 2.6992]	[0.1105, 2.0321]	[0.3044, 1.2558]	[0.0151, 3.0478]	[0.3551, 3.3521]

Decline phase					
**Parameters**	***q***	**alpha**	**Weibull shape**	**Weibull scale**	**Mean longevity (Myrs)**
mean	8.4253	0.3485	**1.488**	3.3262	3.026
median	8.4544	0.3478	**1.4776**	3.3111	3.01
95% HPD	[7.0881, 9.6823]	[0.2946, 0.4036]	**[1.0464, 1.9301]**	[2.6261, 4.0946]	[2.4248, 3.6549]
