# Supplementary material for: Dinosaur biodiversity declined well before the asteroid impact, influenced by ecological and environmental pressures
Source: Nat Commun. 2021 Jun 29;12:3833. doi: 10.1038/s41467-021-23754-0 (PMC8242047; doi:10.1038/s41467-021-23754-0)
Supplement: Supplementary file 3 — Reporting Summary [file 41467_2021_23754_MOESM3_ESM.pdf]

nature research

Corresponding author(s): Fabien L. Condamine

Last updated by author(s): May 5, 2021

Reporting Summary

Nature Research wishes to improve the reproducibility of the work that we publish. This form provides structure for consistency and transparency in reporting. For further information on Nature Research policies, see Authors & Referees and the Editorial Policy Checklist .

Statistics

For all statistical analyses, confirm that the following items are present in the figure legend, table legend, main text, or Methods section.

n/a	Confirmed	
☐	☒	The exact sample size (*n*) for each experimental group/condition, given as a discrete number and unit of measurement
☒	☐	A statement on whether measurements were taken from distinct samples or whether the same sample was measured repeatedly
☒	☐	The statistical test(s) used AND whether they are one- or two-sided<br>*Only common tests should be described solely by name; describe more complex techniques in the Methods section.*
☐	☒	A description of all covariates tested
☐	☒	A description of any assumptions or corrections, such as tests of normality and adjustment for multiple comparisons
☐	☒	A full description of the statistical parameters including central tendency (e.g. means) or other basic estimates (e.g. regression coefficient) AND variation (e.g. standard deviation) or associated estimates of uncertainty (e.g. confidence intervals)
☒	☐	For null hypothesis testing, the test statistic (e.g. *F*, *t*, *r*) with confidence intervals, effect sizes, degrees of freedom and *P* value noted<br>*Give P values as exact values whenever suitable.*
☐	☒	For Bayesian analysis, information on the choice of priors and Markov chain Monte Carlo settings
☒	☐	For hierarchical and complex designs, identification of the appropriate level for tests and full reporting of outcomes
☒	☐	Estimates of effect sizes (e.g. Cohen's *d*, Pearson's *r*), indicating how they were calculated

Our web collection on statistics for biologists contains articles on many of the points above.

Software and code

Policy information about availability of computer code

Data collection	No specific tool was used to collect the data.
Data analysis	All analyses were performed using PyRate version 3.0 (built 20200406). PyRate is a Bayesian model with MCMC algorithm to estimate origination, extinction and preservation rates from fossil occurrence data. We have provided the command lines to run all the models we have performed with the fossil datasets (https://doi.org/10.6084/m9.figshare.14169575.v1).

For manuscripts utilizing custom algorithms or software that are central to the research but not yet described in published literature, software must be made available to editors/reviewers. We strongly encourage code deposition in a community repository (e.g. GitHub). See the Nature Research guidelines for submitting code & software for further information.

Data

Policy information about availability of data

All manuscripts must include a data availability statement. This statement should provide the following information, where applicable:

- Accession codes, unique identifiers, or web links for publicly available datasets
- A list of figures that have associated raw data
- A description of any restrictions on data availability

We collected fossil data for the six dinosaur families for which specimen occurrences are available by starting with a raw dataset obtained from the Paleobiology Database (through Fossil Works: http://fossilworks.org/) and completed with exhaustive check of the literature for taxonomy, species names, and Museum specimen number. All dinosaur data sets to do the analyses are available through the Figshare digital data repository (https://doi.org/10.6084/m9.figshare.14169575.v1).

Field-specific reporting

Please select the one below that is the best fit for your research. If you are not sure, read the appropriate sections before making your selection.

- [ ] Life sciences
- [ ] Behavioural & social sciences
- [x] Ecological, evolutionary & environmental sciences

For a reference copy of the document with all sections, see nature.com/documents/nr-reporting-summary-flat.pdf

Ecological, evolutionary & environmental sciences study design

All studies must disclose on these points even when the disclosure is negative.

Study description	We assessed whether the dominant dinosaur clades of the Late Cretaceous were in decline before the asteroid impact. We further tested the relationship between speciation and/or extinction rates and key environmental variables that represent past fluctuations of the Cretaceous environment. The study focused on six non-avian dinosaur families.
Research sample	The research sample included all species of six non-avian dinosaur families for which we could obtain fossil occurrence data such that they could be included in a comprehensive fossil dataset of Late Cretaceous dinosaurs. The sample was global in scope, and there were no data exclusions.
Sampling strategy	Sample size was determined by availability of fossil data for dinosaurs. We used all available data in our study. The sample size used in our study corresponds to the majority of published fossil-based diversification studies. Moreover, the extent of speciation and extinction rates heterogeneity across dinosaurs as inferred using PyRate is sufficiently great as to enable us to detect drivers of diversification and/or extinction.
Data collection	Fossil occurrence data were extracted from public fossil databases (Paleobiolgy Database, Fossil Works). Ecological data for dinosaurs (e.g. diet) were extracted from the litterature.
Timing and spatial scale	Timing scale (scope) of the study is the Late Cretaceous (i.e. from 100.5 milllion years ago to 66 million years ago). The spatial scale is global, although the sampling is biased with the Northern Hemisphere being more sampled (more fossil localities). However, this question is generally not applicable to our study.
Data exclusions	No data were excluded.
Reproducibility	Our data are not experimental and experiments were thus not replicated. However, we used multiple distinct statistical tests (e.g. environment-dependent diversificaiton models, multivariate birth-death models) and all approaches yielded concordant results.
Randomization	We did not perform an experiment and there was thus no group allocation. We used all species of the six dinosaur families for which (1) fossil occurrence data were available, and (2) diet data were available (herbivorous and carnivorous). There was no further group partitioning of data beyond the natural groupings associated with clade and diet membership.
Blinding	Blinding was not relevant to our study, because all available data were used (our study did not perform an experiment).

Did the study involve field work? [ ] Yes [x] No

Reporting for specific materials, systems and methods

We require information from authors about some types of materials, experimental systems and methods used in many studies. Here, indicate whether each material, system or method listed is relevant to your study. If you are not sure if a list item applies to your research, read the appropriate section before selecting a response.

Materials & experimental systems

n/a	Involved in the study	
[x]	[ ]	Antibodies
[x]	[ ]	Eukaryotic cell lines
[ ]	[x]	Palaeontology
[x]	[ ]	Animals and other organisms
[x]	[ ]	Human research participants
[x]	[ ]	Clinical data

Methods

n/a	Involved in the study	
[x]	[ ]	ChIP-seq
[x]	[ ]	Flow cytometry
[x]	[ ]	MRI-based neuroimaging

Palaeontology

Specimen provenance	No new specimens are provided. The fossil datasets are made through a compilation of the fossil data first downloaded from the Paleobiology Database (through Fossil Works: http://fossilworks.org/).
Specimen deposition	All specimens have already been deposited into Museum institutions, and in online database such as Paleobiology Database (https://paleobiodb.org/) and Fossil Works (http://fossilworks.org/).
Dating methods	No new dates are provided. Ages of fossil occurrences come from the stratigraphic data bearing the fossils. Chronostratigraphic data come from the International Commission on Stratigraphy and sensu ,Gradstein, F.M, Ogg, J.G., Schmitz, M.D., et al., 2012, The Geologic Time Scale 2012: Boston, USA, Elsevier, https://doi.org/10.1016/B978-0-444-59425-9.00004-4.

☒ Tick this box to confirm that the raw and calibrated dates are available in the paper or in Supplementary Information.
